# Experimental Study of an Enhanced Phase Change Material of Paraffin/Expanded Graphite/Nano-Metal Particles for a Personal Cooling System

**DOI:** 10.3390/ma13040980

**Published:** 2020-02-22

**Authors:** Chuyuan Ma, Ying Zhang, Xianfeng Chen, Xiande Song, Kaixuan Tang

**Affiliations:** 1 School of Safety Science and Emergency Management, Wuhan University of Technology, Wuhan 430070, China; docmcy@whut.edu.cn (C.M.); yzhang@whut.edu.cn (Y.Z.); cxf618@whut.edu.cn (X.C.); xixixiande@163.com (X.S.); 2 Guangzhou Expressway co. LTD, Guangzhou Transportation Investment Group, Guangzhou 510288, China

**Keywords:** composite phase change material, personal cooling system, thermal conductivity, heat storage, nano-metal particle

## Abstract

A composite phase change material (PCM) was prepared by incorporating paraffin (PA) with expanded graphite (EG) and nano-metal particles to improve the thermal conductivity and reduce the leakage performance of PA once it melts and, consequently, develop a more efficient PCM for a personal phase change cooling system. A series of experiments was carried out by a scanning electron microscope, a differential scanning calorimeter, a hot-disk thermal analyzer, and leakage tests on the composite PCM with various mass fractions of EG and metals (i.e., Cu, Al, Ni, and Fe). Through comprehensive consideration of the thermal conductivity, leakage, and homogeneity, a composite PCM with the optimal proportion (PA-EG11%-Cu1.9%) was screened out. Its thermal conductivity was significantly improved nine times, while the phase change enthalpy showed a minimal decrease. In addition, the relationships of the composite PCM with its temperature and density were systematically investigated. The experimental results are important for determining the proper package density of PCM for application into a personal cooling system because its weight is crucial for the system design and benefits the performance comparison of various PCMs prepared under various conditions. Lastly, the heat storage efficiency of the PA–EG–Cu material was investigated using heat storage tests. Cooling performance clearly improved compared to the PCM without nano-particles added.

## 1. Introduction

Heat stress caused by operations in high-temperature environments (including military, automotive, iron, steel, metallurgical plants, glass manufacturing, and mining [1]) has become a major threat to human health [2]. Thermal fainting can endanger human life when severe. With the thermal protection of workers in high-temperature environments, a personal cooling system is a good countermeasure for heat stress [3].

Cooling clothing includes liquid cooling clothing [4], gas cooling clothing [5], and phase change material (PCM) cooling [6]. The cooling medium of gas cooling clothes is air, which is composed of basic clothing and air compressors. Cold air is piped to various parts of the human body for cooling. Liquid cooling clothes are composed of a pre-cooling device and basic clothing. The cooling medium of this type of cooling clothing are an ice water mixture, water, water and vinyl glycol below 0 °C, etc. [7]. Cooling takes the form of frozen liquid flowing to the entire human body through a pipeline network, which has good ventilation and high air-conditioning efficiency [8]. These two kinds of cooling clothes have complicated structures and difficult designs, which cause a major inconvenience when dressing the human body. Phase change cooling clothing is a kind of cooling clothing that uses PCM as a cold source to cool the human body. It is a passive cooling process that uses PCM to absorb heat in the process of changing physical conditions in high-temperature environments. When the temperature decreases, the absorbed heat is released [9]. Compared with gas cooling and liquid cooling clothing, phase change cooling clothes have the advantages of a simple structure, low pollution, low cost, low demand on the environment, and high application value. Chan et al. evaluated the effectiveness and practicality of two commercially available cooling vests in four industries [10,11,12]. Although the cooling effects of the vests were confirmed, workers were still unsatisfied with them because of their short cooling time and heavy weight. Thus, the authors developed a newly designed hybrid cooling vest with a total weight of 1.26 kg for construction workers using PCM and a ventilation fan. A field study was conducted in the construction sites of Hong Kong to evaluate its effectiveness and practicality in the construction industry [13,14]. The field study found that this cooling vest significantly improved the comfort by approximately 1.23 (p < 0.001) in a seven-point scale. Moreover, 91% of the subjects preferred this HCV as a cooling measure during rest [13]. However, the PCM used in the HCV is sodium sulfate decahydrate, which has low thermal conductivity and a risk of melting leakage. Therefore, a composite phase change cooling material with high thermal conductivity and low leakage that is more suitable for cooling clothes must be developed, which is also the purpose of this article.

Five principles should be considered when choosing the PCM used in cooling clothing: safety: environmental protection, economic practicability, high efficiency, and reliability of the materials. Paraffin (PA) [15] has the characteristics of high energy storage density, stable chemical properties, and low price and has been widely used in the low-temperature and medium-temperature fields [16,17,18,19]. However, the low thermal conductivity and easy leakage of PA limit its application in personal cooling systems. To overcome the problem of low thermal conductivity, numerous studies were carried out to prepare the composite PCM with high thermal conductivity material [20,21,22], such as embedding dispersing metallic particles into pure PCM [23,24,25], blending PCM with nanoparticles [26,27], using differently configured finned tubes in storage units [28,29,30], and impregnating PCM into a high thermally conductive material with a porous structure [31,32]. Py. Xavier et al. [33] added expanded graphite (EG) to PA, which clearly improved its thermal conductivity. Sari and Karaipekli [34] measured the thermal conductivity of the PA-EG composite PCM with different expansion graphite contents. However, the improvement of thermal conductivity by increasing the proportion of EG [35,36,37,38,39,40,41,42] decreased the phase change enthalpy as well as the latent heat capacity. Many studies on composite PCM have been carried out by previous researchers to improve the thermal conductivity of PA, but the results differed greatly from one another, and the density of the composite PCM in different studies was different, which makes it difficult to compare the results with one another. The effect of temperature on thermal conductivity is also critical. Therefore, the thermal conductivity of composite PCM with different densities and temperatures should be investigated.

In this study, EG was used as an adsorption material, and PA was shaped by its network structure to prevent leakage during its use. The addition of high thermally conductive nano-metals increased the thermal conductivity of the PA-EG-based composite PCM, and the distributions of nano-metal copper (Cu), aluminum (Al), iron (Fe), and nickel (Ni) particles were selected. Analysis and comparison of scanning electron microscope (SEM), differential scanning calorimeter (DSC), and thermal conductivity of composite PCM with different nanoparticles were carried out to select the best nano-metal particles for improving PA-EG performance. Thermal storage experiments verified that the addition of nano-metal particles could improve the thermal storage efficiency of composite PCM. The effect of temperature and density on the composite PCM was investigated, and the volume expansion rates were compared before and after the phase transitions of different densities. The volume expansion ratio was also compared before and after the phase transition of different densities, and the package density of composite PCM in the cooling suit was determined. Thus, a high-efficiency cooling PCM that could be applied to cooling clothes was prepared.

## 2. Materials and Methods

### 2.1. Experimental Materials and Instruments

The base organic PCM is PA (paraffin)(Guangzhou Wengjiang Chemical Reagent Co., Ltd, Guangzhou, China, purity ≥ 98.0%), and its parameters are shown in Table 1.

The number of EG mesh is 50, the purity is more than 99%, the rate of expansion is 600 mL/g, nano-Cu, nano-Al, nano-Fe, and nano-Ni (Changsha Tianjiu Metal Materials Co., Ltd, Changsha, China).

The instruments used in the experiment include the following: Electronic precision balance (Precision Scientific Instruments Shanghai Co., Ltd, Shanghai, China), digital display constant-temperature water bath pot (Crystal Technology & Industries, Inc, Dallas, TX, USA), constant-temperature drying box (Guangdong Lab Companion Ltd, Guangdong, China), ultrasonic oscillator (Skymen Cleaning Equipment Shenzhen, Shenzhen, China), and refrigerator (Haier, Wuhan, China), DSC (Netz Scientific Instruments Trading (Shanghai) Co., Ltd., Shanghai, China), hot-disk thermal conductivity tester (K-analysis Instrument Trading Shanghai, Shanghai, China), SEM (Hitachi High-Tech Company, Beijing, China), and a data acquisition instrument (Hitachi (Shanghai) Trading Company, Shanghai, China).

### 2.2. Preparation of PA/EG/Nano-Metal Composite PCMs

**Figure 1:**
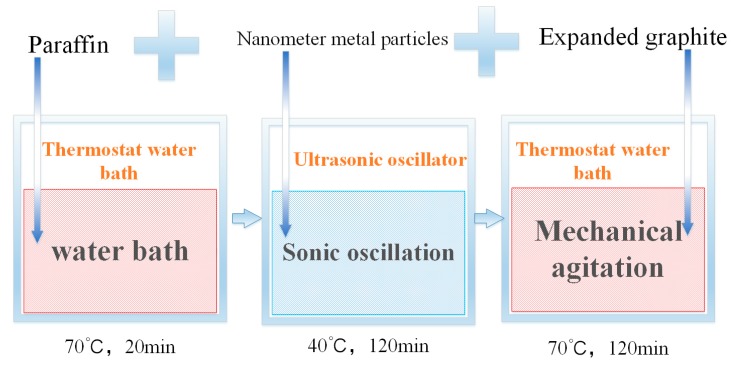
Preparation of composite phase change material (PCM).

The density of nano-metals is relatively high, and the direct use of melt blending will cause the phenomena of sinking and agglomeration of nano-metal particles. This study uses the two-step method to prepare a PA/EG/nano-metal composite PCM. Figure 1 shows the experimental process. First, the PA is melted in a water bath at 70 °C. Next, the nano-metal is added into an ultrasonic shaker at 40 °C and then oscillated for 2 h. Lastly, EG is added with mechanical stirring for 2 h to obtain a PA/EG/nano-metal composite PCM. The prepared samples are shown in Table 2.

### 2.3. Adsorption Experiments

**Figure 2:**
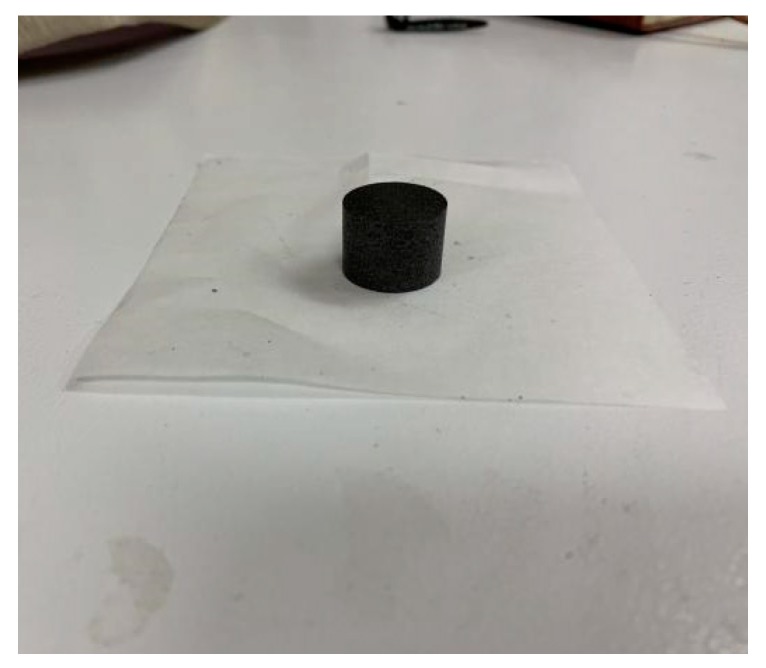
Paraffin-expanded graphite (PA-EG) mold.

EG with a loose porous mesh structure has good adsorption, radiation resistance, and flame retardancy. A PA-EG composite PCM was produced by absorbing the liquid PA into the EG, which was added at ratios of 5%, 8%, 11%, and 15%. Figure 2 is a cylindrical model with a bottom surface diameter of 10 mm and a height of 10 mm after mold treatment. The PA-EG model was placed in a 45 °C incubator for 3 h, and the leakage of PA on the filter paper was observed.

### 2.4. Performance Characterization of Composite PCM

SEM: Morphology analysis of composite PCMs was performed by SEM. The experimental instrument used in the SEM was a Hitachi SU8010 with a resolution of 1.0 nm (15 kV), 1.4 nm (1 kV, WD = 1.5 nm, deceleration mode), and 2.0 nm (1 kV, WD = 1.5 nm, normal mode). The electron microscope has a magnification of 30×–8,000,000×. The experiment can be used to characterize the morphology, particle size, and dispersion of the sample. The homogeneity of the composite material was investigated after the addition of different nano-metals at different proportions to determine whether the nano-metal particles have serious agglomeration.

DSC experiment: Take a mg sample in the N_2_ atmosphere. Set the initial temperature to −10 °C, increase to 100 °C at a heating rate of 5 °C/min, and then drop it back to −10 °C at a cooling rate of 5 °C/min. The phase change temperature and enthalpy of the composite PCMs with different metals and different metal proportions were studied.

The thermal conductivity of different composites was measured using a hot-disk thermal property tester. The model of probe polyimide film was 7577, the size was r = 2.001 mm, repeatability was <1%, accuracy was <3%, and temperature was 20 °C. The thermal conductivity of the composite PCM was tested at different temperatures and densities, and the effects of temperature and density on the thermal conductivity of the composite PCM were investigated.

### 2.5. Thermal Storage Experiment of Composite PCM

**Figure 3:**
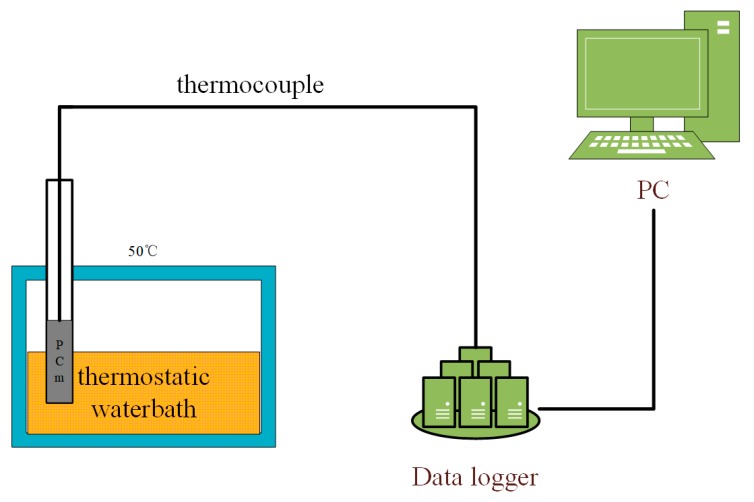
Heat storage experiment.

The heat storage experiment verified the heat storage efficiency after the addition of nano-metal particles. Figure 3 shows the experimental process. The prepared composite PCM is placed in a refrigerator for freezing treatment and then placed in a 50 °C thermostatic water bath. The data acquisition system measures the temperature rise of the sample through a thermocouple.

### 2.6. Thermal Cycle Experiment of Composite PCM

Place the prepared composite PCM into a refrigerator at −10 °C to freeze. Next, put the composite PCM in a water bath at 50 °C and leave it for 0.5 h. After the phase change is complete, place the composite PCM in a refrigerator at −10 °C to freeze, repeat the process, and weigh the composite PCM five times per cycle at 100 cycles.

### 2.7. Volume Expansion Test of Composite PCM

The composite PCM was pressed into a cylinder with a bottom area of 10 mm and a height of 10 mm. The composite PCM cylinders with different densities were pressed separately, placed into the refrigerator for freezing treatment, and then placed in a 40 °C incubator. After 2 h of constant temperature treatment, the bottom surfaces of the composite PCM with different densities were measured to determine the micrometer changes in diameter and height.

## 3. Results

### 3.1. Optimized Proportion of EG

**Figure 4:**
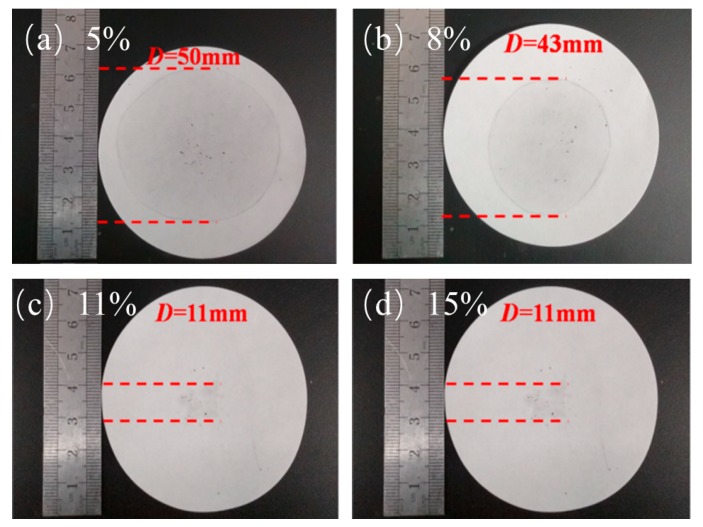
Penetration of different mass fractions: (**a**) EG—5%, (**b**) EG—8%, (**c**) EG—11%, and (**d**) EG—15%.

Figure 4 shows the leakage of PA. The leakage of 5% and 8% EG was serious, which far exceeds the diameter of the material itself and covers almost the entire filter paper. However, with the increase of EG content, the leakage situation was effectively improved. Figure 4c,d reveal that, when the EG content reaches 11% and 15%, their leakage situations are not very different, and the leakage was almost within the size range of the material. It can completely absorb the PA, and the situations for the 11% and 15% EG were the same. This outcome means that, when the EG content reaches 11%, PA can be completely adsorbed. The addition of too much EG has a greater impact on the overall latent heat performance of the material. Hence, the optimal addition amount is 11%.

### 3.2. Effects of Spacing on System Thermal Performance

**Figure 5:**
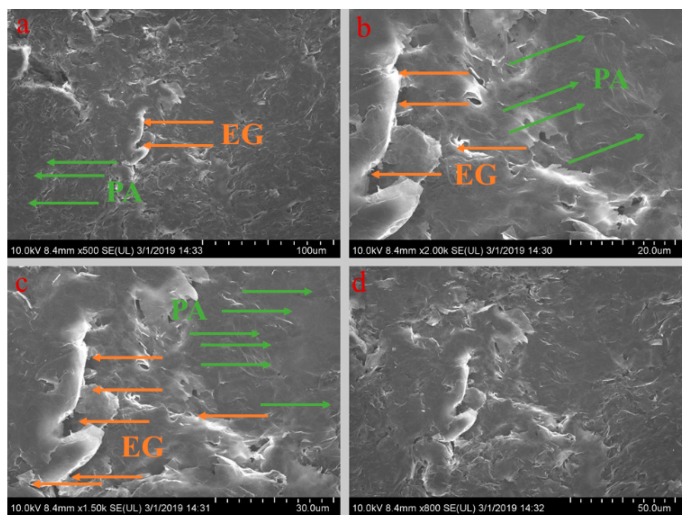
SEM (scanning electron microscope) analysis of PA-EG: (**a**) 500× magnification, (**b**) 2000× magnification, (**c**) 1500× magnification, and (**d**) 800× magnification.

**Figure 6:**
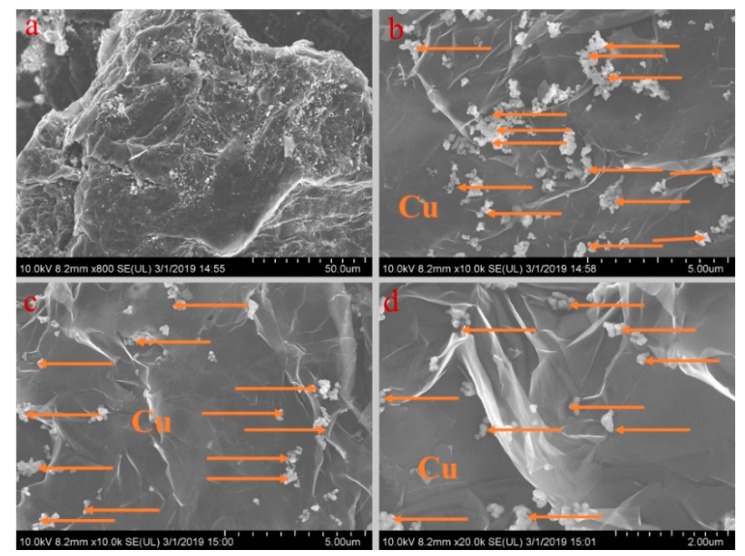
SEM analysis of PA-EG-Cu: (**a**) 800× magnification, (**b**) 10,000× magnification, (**c**) 10,000× magnification, and (**d**) 20,000× magnification.

**Figure 7:**
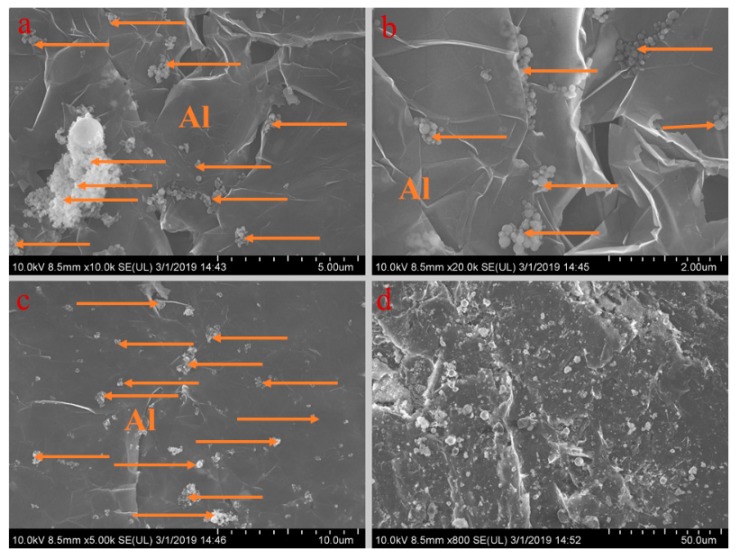
SEM analysis of PA-EG-Al: (**a**) 10,000× magnification, (**b**) 20,000× magnification, (**c**) 5000× magnification, and (**d**) 800× magnification.

**Figure 8:**
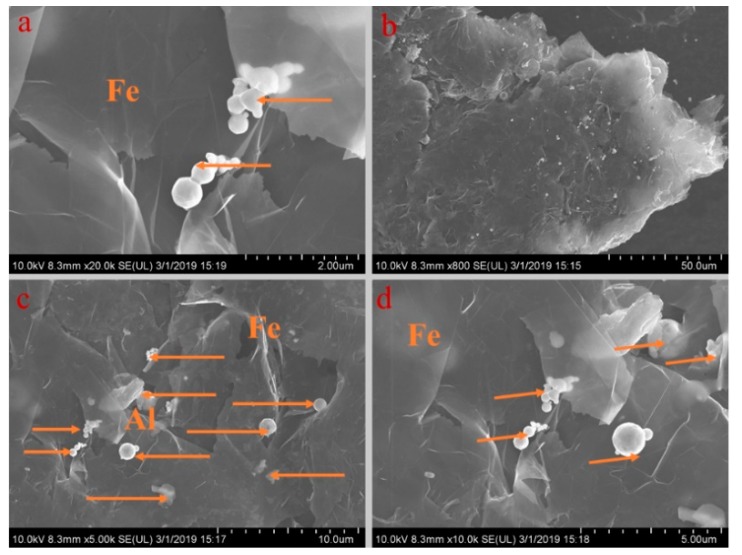
SEM analysis of PA-EG-Fe: (**a**) 20,000× magnification, (**b**) 800× magnification, (**c**) 5000× magnification, and (**d**) 10,000× magnification.

**Figure 9:**
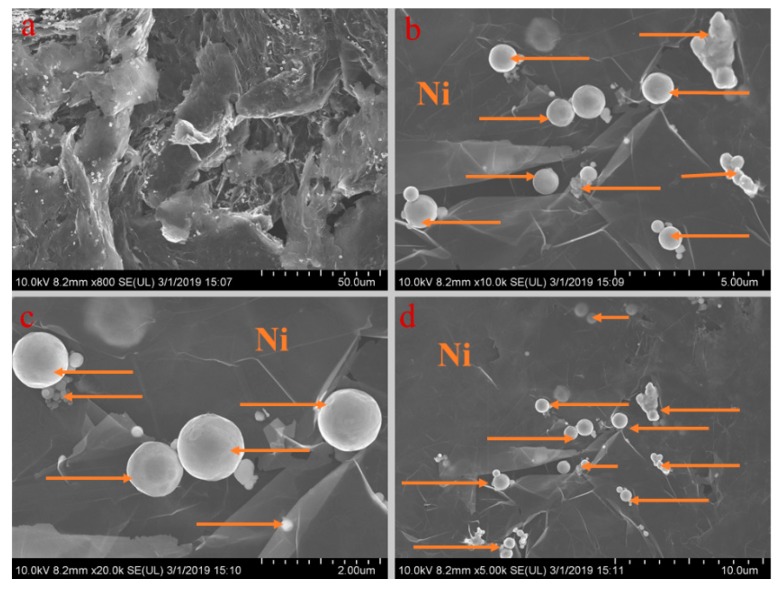
SEM analysis of PA-EG-Ni: (**a**) 800× magnification, (**b**) 10,000× magnification, (**c**) 20,000× magnification, and (**d**) 5000× magnification.

Figure 5, Figure 6, Figure 7, Figure 8 and Figure 9 show the morphologies of PA-EG, PA-EG-Cu, PA-EG-Al, PA-EG-Fe, and PA-EG-Ni. The worm-like microstructure of EG is filled with PA and nano-metal particles, which have a good supporting effect on PA and the nano-metal particles. From observing the microstructure of composites with different nano-metal particles, it can be concluded that the addition of nano-Cu particles and the composite material of nano-Al particles has a relatively uniform distribution in the composite material system. The content in a unit volume is also larger than the other two kinds of nano-metal particles where no agglomeration of nano-Cu and nano-Al are observed after composition with PA-EG. The result indicates that the nano-Cu and nano-Al particles have good compatibility with PA-EG.

### 3.3. DSC (differential scanning calorimeter) Analysis

**Figure 10:**
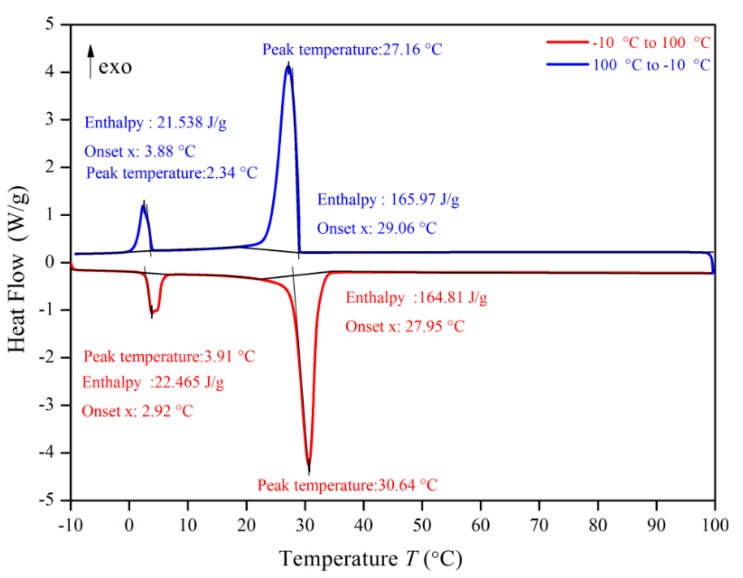
Differential scanning calorimeter (DSC) analysis of PA.

**Figure 11:**
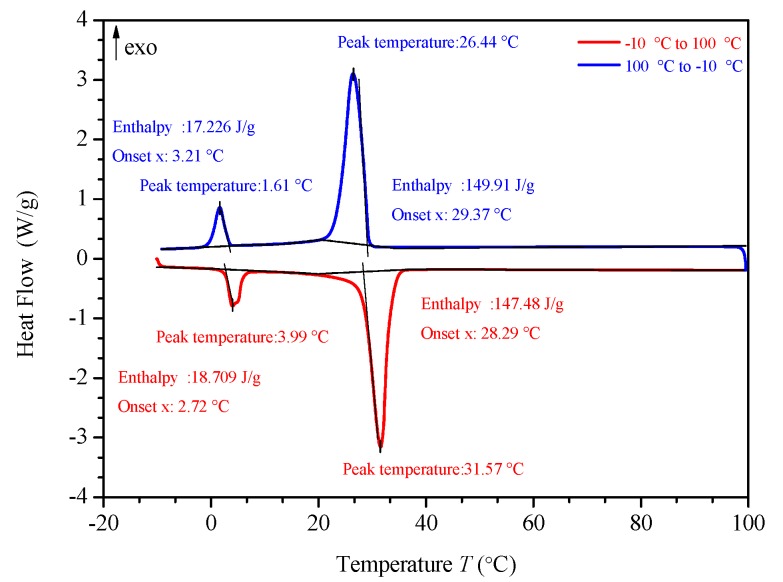
DSC analysis of PA-EG.

**Figure 12:**
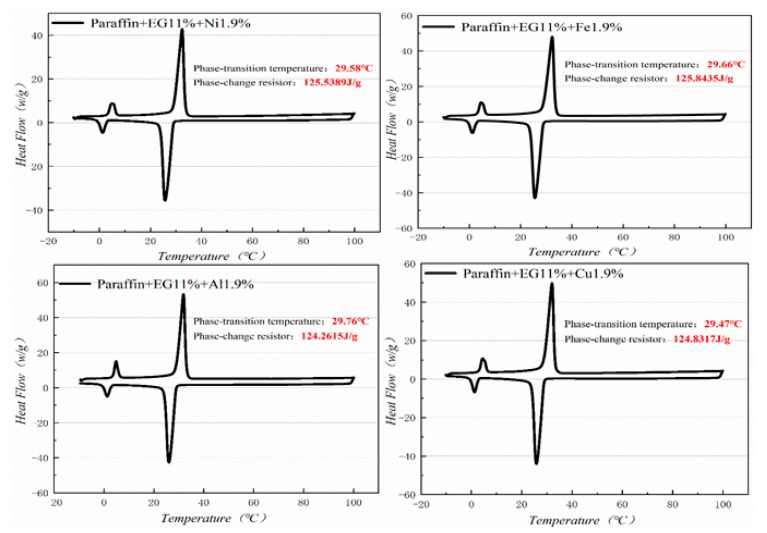
DSC analysis of PA-EG-nano-metal particles.

**Figure 13:**
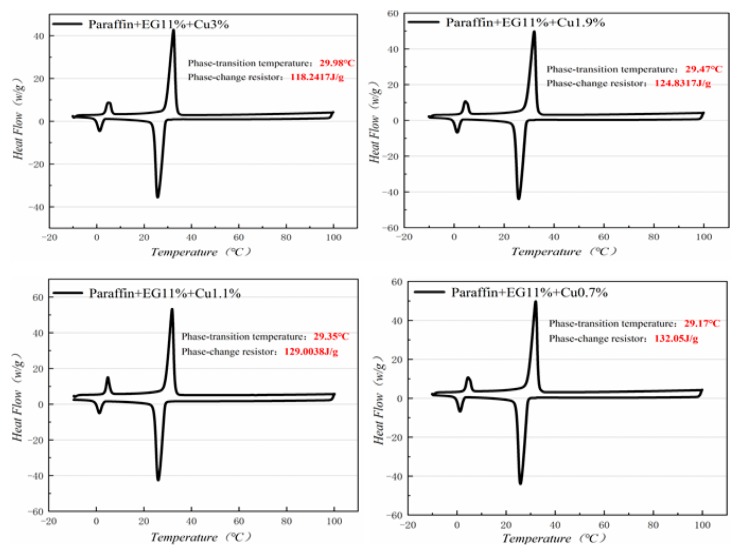
DSC with different mass fractions analysis of PA-EG-Cu.

**Figure 14:**
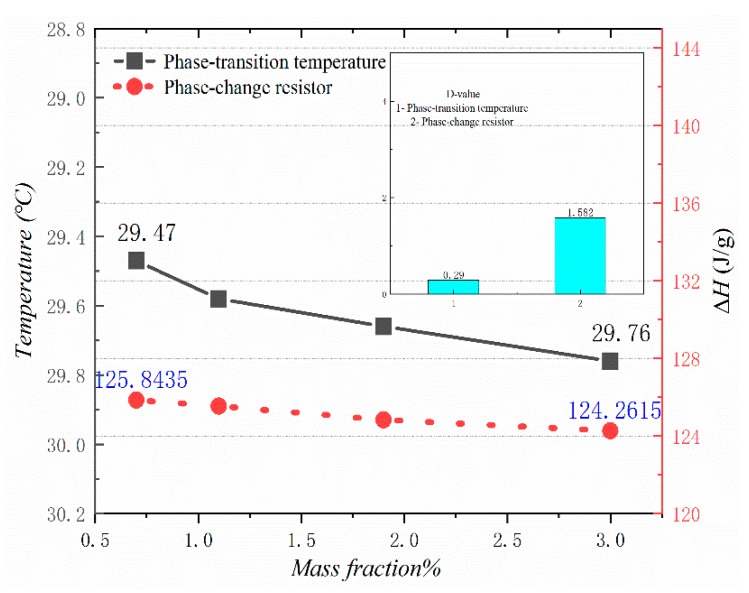
Comparison of the phase change temperature and phase change enthalpy.

**Figure 15:**
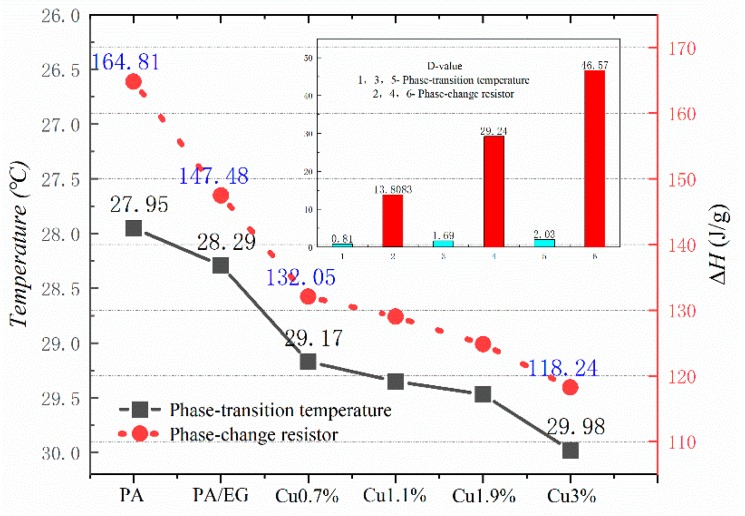
Comparison of the phase change temperature and phase change enthalpy.

Figure 10, Figure 11, Figure 12 and Figure 13 show the DSC curves of PA, PA-EG, and composite PCM with different metals and different addition ratios. Figure 14 reveals the comparison of the phase transition temperature and phase transition enthalpy of the composite PCM with different metals. Figure 15 reveals the comparison of the phase transition temperature and phase transition enthalpy of the composite PCM with different proportions. Compared with PA-EG and PA, the phase transition temperature of the composite PCM with nano-metal particles increased by 1.69 °C and 2.03 °C, respectively. The ideal temperature of the human body is at approximately 30 °C and increasing amplitude has no effect on the application of composite PCM. Compared with PA-EG and PA, the minimum value of the phase change enthalpy of PA-EG-Cu decreased by 29.24 and 46.57 J/g, respectively. The enthalpy of phase change and the temperature of the phase change of the composite PCM have little effect on different metal particles. Different addition ratios have almost no effect on the phase transition temperature of the composite PCM, but have a greater effect on the phase change enthalpy, with a difference of 13.81 J/g.

### 3.4. Thermal Conductivity

**Figure 16:**
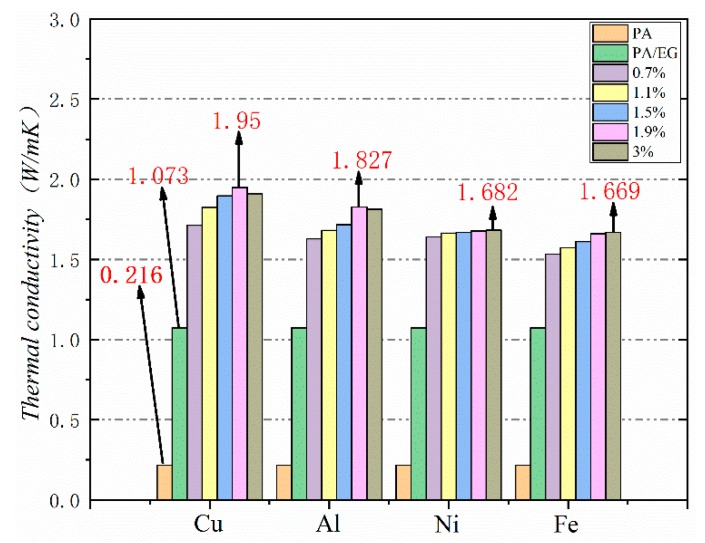
Thermal conductivity of different nano-metals with different mass fractions.

Thermal conductivity is a crucial parameter for PCM because it reflects the material’s heat transfer rate. The higher the thermal conductivity is, the faster the heat absorption will be. Heat absorption, in turn, can increase the efficiency of PCM in practical applications. In this study, different metal particles were added as thermal conductivity enhancers to improve the thermal conductivity of PA–EG-based composite PCM. The test results are shown in Figure 16. The thermal conductivity of PA is very low at 0.216 W/(m·K), while the thermal conductivity of PA-EG is 1.073 W/(m·K), which is an increase of about five times. The growth range is approximately 7.7–9 times when nano-metal particles are added. Four nano-metal particles have different effects on the thermal conductivity of composite PCM. Adding nano-Cu particles works best. EG and nano-metal particles have an enhanced effect on the thermal conductivity of composite PCM while nano-metal particles have a much smaller proportion than EG. When the thermal conductivity is increased, the effect on the phase change enthalpy of the composite PCM is reduced.

**Figure 17:**
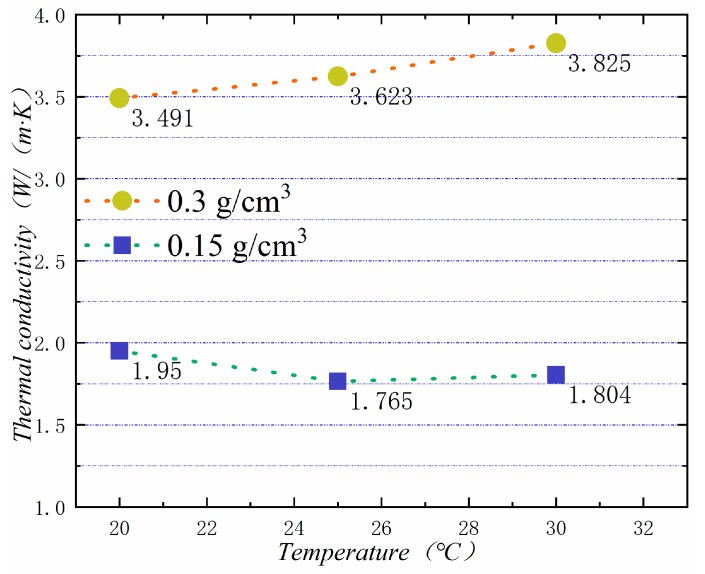
Thermal conductivity at different temperatures.

In general, the higher the temperature is, the higher the thermal conductivity of the material is. Figure 17 shows the thermal conductivity of PA-EG-Cu at different temperatures. The measured densities are 0.15 and 0.3 cm^3^, and the thermal conductivity is linearly related to temperature.

### 3.5. Heat Storage Analysis

**Figure 18:**
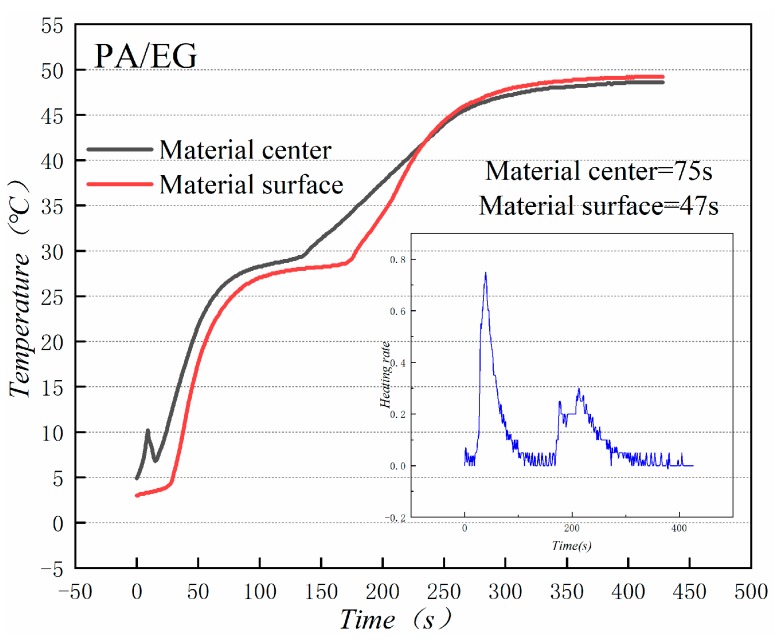
Heat storage curve of PA-EG.

**Figure 19:**
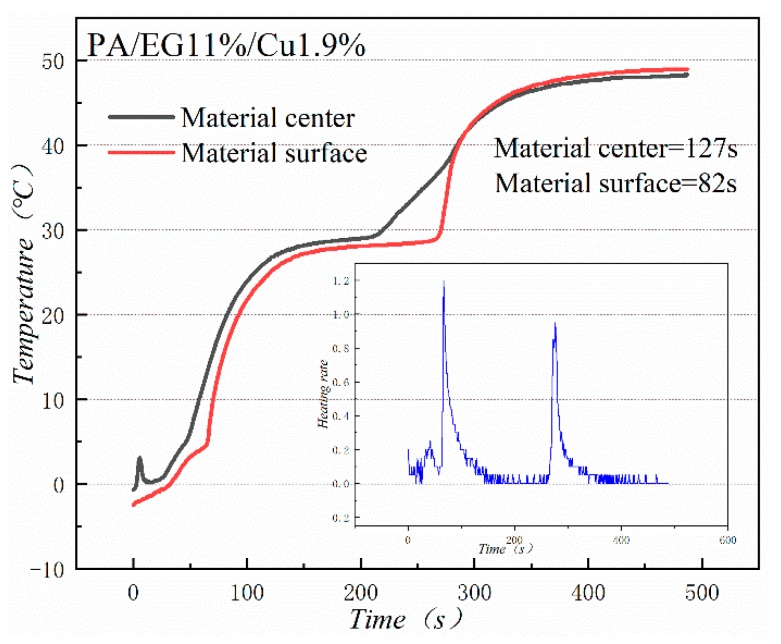
Heat storage curve of PA-EG-Cu.

Figure 18 and Figure 19 illustrate the heat storage curves of PA-EG and PA-EG-Cu, respectively. Two thermocouples were set up during the experiment with one at the surface of the composite PCM and the other at the center of the composite PCM. The heating process selects the time of 5 °C to reach the phase change temperature. Without the addition of nanoparticles, the phase transition time of the surface of the material is 78 s, the phase transition time of the material center is 98 s, and the phase transition process times are 47 and 75 s, respectively. When nano-metal particles are added, the phase transition time of the surface of the material is 46 s, the phase transition time of the material center is 48 s, and the phase transition process times are 82 s and 127 s, respectively. Clearly the addition of nano-sized metallic Cu particles can cause the composite PCM to reach the phase change process faster, which significantly increases the heating rate, and lengthens the duration of the phase change.

### 3.6. Determination of Package Density

**Figure 20:**
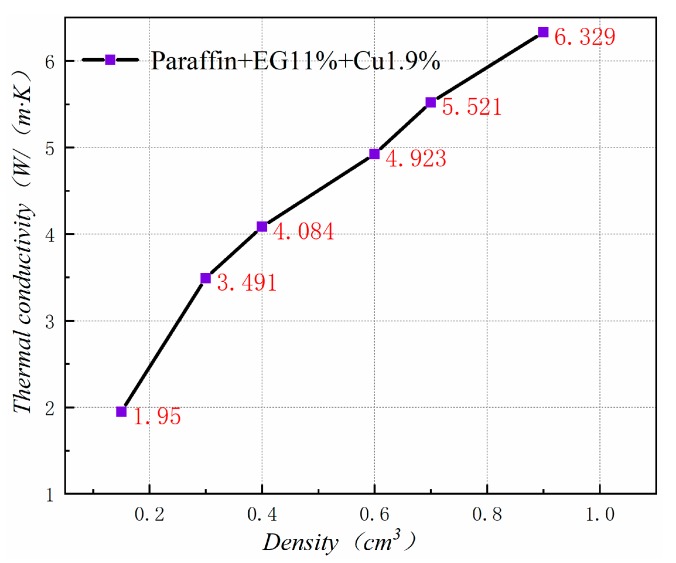
Thermal conductivity at different densities.

Figure 20 shows the thermal conductivity of PA-EG-Cu at different densities. Thermal conductivity has a linear relationship with density. As the density of the composite material increases, the thermal conductivity of the composite material increases. This outcome has great significance for the production and application of composite PCM.

Table 3 shows the effect of density on the volume expansion of composite PCM after heating. When the compressed density is below 0.6 cm^3^, the phase change of the composite PCM can be well supported in the EG, and the volume will not change. When the compressed density is greater than 0.6 cm^3^, the worm structure in the EG is compressed severely due to the compression force being too large. Therefore, a volume change occurs during the phase transition. At a density of 0.9 cm^3^, the volume expands by 66%. Given the thermal conductivity of the composite PCM and the volume expansion after heating, the package density should be 0.6 cm^3^ for application in the cooling suit.

### 3.7. Thermal Stability Analysis

**Figure 21:**
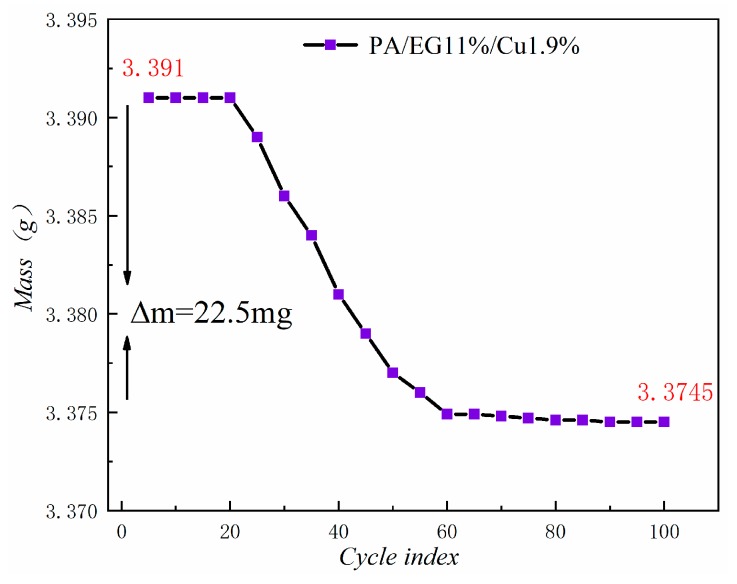
Mass loss curve.

Figure 21 shows the mass loss of the composite PCM after 100 cycles. The figure reveals that the magnitude of the mass loss is only 0.66%. In addition, the decrease becomes increasingly smaller in the future and is basically a stable state. Thus, the thermal stability of this composite PCM is good.

## 4. Conclusions

1 When the proportion of EG is 11%, almost no leakage of molten PA occurs. The reticular structure of EG can effectively suppress the agglomeration of nano-metal Cu and Al, which makes it evenly distributed and well compounded in the PA-EG-based composite PCM.
2 A screening of the different nano-metal particles and different mass fractions added reveals that PA-EG (11%)-Cu (1.9%) is the best-performing cooling PCM. As this composite PCM substantially improves the thermal conductivity, its phase change enthalpy decreases very little, and the phase change temperature satisfies the most suitable temperature range of the human body. Thus, it can be well applied in cooling clothes.
3 The comparison of heat storage analysis between screened PA-EG-Cu and PA-EG indicates that the heat storage speed of the composite PCM with the addition of nano-metal Cu particles is accelerated and the heat storage capacity is strengthened. The thermal stability of PA-EG-Cu is analyzed, and its mass loss tends toward a stable state in the later stage. Thus, it has a high life in the application process.

## Figures and Tables

**Table 1:** Alkane parameters.

Name	Molecular Formula	Melting Temperature (°C)	Latent Heat (J/g)
N-octadecane	C_18_H_38_	28.2	242.67
N-eicosane	C_20_H_42_	36.6	246.86

**Table 2:** Composition of the composite PCMs.

Samples	Composition (wt %)	Samples	Composition (wt %)
PCM1	PA	PCM12	PA–EG11-Al3
PCM2	PA–EG11	PCM13	PA–EG11-Fe0.7
PCM3	PA–EG11-Cu0.7	PCM14	PA–EG11-Fe1.1
PCM4	PA–EG11-Cu1.1	PCM15	PA–EG11-Fe1.5
PCM5	PA–EG1-+Cu1.5	PCM16	PA–EG11-Fe1.9
PCM6	PA–EG11-Cu1.9	PCM17	PA–EG11-Fe3
PCM7	PA–EG11-Cu3	PCM18	PA–EG11-Ni0.7
PCM8	PA–EG11-Al0.7	PCM19	PA–EG11-Ni1.1
PCM9	PA–EG11-Al1.1	PCM20	PA–EG11-Ni1.5
PCM10	PA–EG11-Al1.5	PCM21	PA–EG11-Ni1.9
PCM11	PA–EG11-Al1.9	PCM22	PA–EG11-Ni3

**Table 3:** Volume change tables for different densities.

Experiment No.	Density (g/cm^3^)	Bottom Diameter (mm)	Height (mm)	Volume (cm^3^)
1	0.15	10	10	0.785
2	0.3	10	10	0.785
3	0.4	10	10	0.785
4	0.6	10	10	0.785
5	0.7	10.32	10.12	0.846
6	0.8	10.96	10.64	1.003
7	0.9	12.37	10.87	1.306
